# Efficacy of antimicrobial coated orthopaedic implants on the prevention of periprosthetic infections: a systematic review and meta-analysis

**DOI:** 10.7150/jbji.44839

**Published:** 2020-06-27

**Authors:** Olga D. Savvidou, Angelos Kaspiris, Ioannis Trikoupis, George Kakouratos, Stavros Goumenos, Dimitra Melissaridou, Panayiotis J. Papagelopoulos

**Affiliations:** 1 1 st Department of Orthopaedic Surgery, School of Medicine, National and Kapodistrian University of Athens, “ATTIKON” University General Hospital, Athens, Greece.; 2 Laboratory of Molecular Pharmacology/Division for Orthopaedic Research, School of Health Sciences, University of Patras, Patras 26504, Greece.

**Keywords:** antimicrobial, infection, orthopaedic implant

## Abstract

**Introduction:** Implant-associated infections are a major problem in orthopaedic surgery. Local delivery systems of antimicrobial agents on the implant surface have attracted great interest recently. The purpose of this study was to identify antimicrobial coatings currently used in clinical practice, examining their safety and effectiveness in reducing post-operative infection rates.

**Materials and Methods:** A systematic review was conducted in four databases (Medline, Embase, Cochrane, Cinahl) according to the Preferred Reporting Items for Systematic reviews and Meta-analysis (PRISMA) guidelines up to December 2019, using the key words “orthopaedic implant coated”, “coated implant infection”, “silver coating ” and “antibiotic coating”.

**Results:** Seven articles involving 1307 patients (561 with coated implants and 746 controls who were not) comparing the incidence of periprosthetic infections after the application of internal fracture fixation, total arthroplasties and endoprostheses were evaluated. Three different coating technologies were identified: gentamicin coating for tibia nail and total arthroplasties; silver technology and povidone-iodine coating for tumour endoprostheses and titanium implants. Meta-analysis demonstrated that patients who were treated with antimicrobial coated implants presented lower infection rates compared to controls over the seven studies (Q = 6.1232, I2 = 0.00, 95% CI: 1.717 to 4.986, OR: 2.926, Z= 3.949, p<0.001). Subgroup statistical analysis revealed that each coating technique was effective in the prevention of periprosthetic infections (Q = 9.2606, I2 = 78.40%, 95% CI: 1.401 to 4.070, OR: 2.388, Z= 3.200, p<0.001).

**Conclusion:** All technologies were reported to have good biocompatibility and were effective in the reduction of post-operative peri-prosthetic infection rates.

## Introduction

Implant infection is a very severe complication in orthopaedics, resulting in implant failure, osteomyelitis and septic arthritis. The frequency of periprosthetic infections after arthroplasty ranged between 3% and 15%, respectively 1-2. After resection of large bony fragments and application of megaprosthesis, the infection risk increased to 36% 3.

Although various strategies have been developed to eliminate infection risks, such as ultra clean operation rooms, standardised surgical techniques or improved-design prostheses, post-arthroplasty infections remain a serious complication. The main pathogen associated with this problem is *staphylococcus* 4-5. Coagulase-negative *staphylococci* species account for 30-41%, while *Staphylococcus aureus* (*S. aureus*) for 12-39% of all cases 6-7. The increased frequency of methicillin-resistant *S. aureus* (MRSA) isolation in infected implants is associated with a worse overall outcome 8. Bacterial adhesion and proliferation results in biofilm formation, local infection, bone loss and altered activity of the resident bone cells, leading to restriction of prosthesis osseointegration. Furthermore, biofilms a) act as a protective barrier against antibiotics and host defence; b) have a low metabolic rate, making eradication and detachment of the infective agent difficult c) are internalised by osteoblasts, protecting them from the host immune system 9-11.

Systemic antibiotic prophylaxis remains the first line strategy, although several studies have demonstrated that it achieves poor surgical site penetration due to the ischaemic and necrotic nature of post-operative tissue and is accompanied by serious side effects, such as liver or kidney toxicity 12. Current infection prophylaxis concepts emphasise the implant surface interface and the surrounding tissue and focus on local delivery of antimicrobial substances into the implant-surrounding tissues 13. Based on the above notion antimicrobial coating of the implants may provide an efficient method of prevention and treatment of implant-associated infections. Three different coating technologies were identified: gentamicin coating (poly D-, L- lactide matrix and Defensive Antibacterial Coating hydrogel) for tibia nail and total arthroplasties; silver technology and povidone-iodine coating for tumour endoprostheses and titanium implants. However, the literature on the application of antimicrobial coating on orthopaedic implants is limited and does not provide adequate evaluation of the impact of their use on clinical practice. Moreover, most studies do not include multi-centre findings or quantitative synthesis of these results 13.

The aim of our systematic review and meta-analysis was to determine the odd ratio of periprosthetic infections after application of antimicrobial coating techniques compared to controls in orthopaedic surgery and to establish whether there is an association of antimicrobial coating with a decreased or increased risk of periprosthetic infections. We assumed that the application of antimicrobial coating is correlated with a reduced risk of periprosthetic infection in patients undergoing orthopaedic surgery.

## Materials and Methods

### Research Strategy

A systematic computer-based literature review search with predefined criteria was performed on 08 December 2019 according to the Preferred Reporting Items for Systematic Reviews and Meta-Analyses (PRISMA) guidelines in the following databases: PubMed (1947 to present), Web of Science (1900 to present), the Cochrane Database of Systematic Reviews (1992 to present), Embase (1974 to present), Ovid, Google Scholar (early 1900 to present) and the WHO International Clinical Trials Registry platform. The research methodology used a combination of the following terms: “orthopaedic implant coated [All Fields]”, “coated implant infection [All Fields]”, “silver coating [All Fields]” and “antibiotic coating [All Fields]”. The electronic literature search was conducted independently by two authors (ODS, PJP) and an experienced librarian. Moreover, the above two senior authors (ODS and PJP) independently screened the titles and abstracts to identify relevant studies of clinical outcomes and periprosthetic infection complications after the application of antimicrobial coating. If there was a disagreement between them, the final decision was made by the senior author.

### Inclusion criteria and study selection

Studies that compared the outcome and the incidence of periprosthetic infections in patients after the application of an antimicrobial coating technique were identified. For the definition of periprosthetic infection, the criteria of International Consensus Meeting (ICM) and Infectious Diseases Society (IDSA) were used 14. Surgical site infections were defined as the presence of positive local clinical signs of acute inflammation and/or draining sinus requiring further surgery, early debridement, implant removal or unplanned antibiotic treatment. Only full-text articles were eligible for inclusion. Additional inclusion criteria included: a) studies written in English b) comparative studies assessing the application of antimicrobial coating in orthopaedic surgery, c) surveys concerning the application of an antimicrobial-coated implant in human subjects and d) data on the outcome should have been clearly given to each patient. Publication date limitations were not set.

Studies that did not include comparative results or written in a language other than English were excluded. Case reports, reviews, letters to the editor, expert opinions articles with insufficient details about the type of intervention, the clinical outcome regarding post-operative infection rates and surveys with non-obtainable data were excluded. Research based only on *in vitro* or *in vivo* animal models results was also excluded.

### Data extraction

Two reviewers (ODS and PJP) examined all the identified studies. All data of each study was asse mbled in a Microsoft Excel spreadsheet, classified per orthopaedic intervention and type of coated prosthesis. Characteristics extracted from clinical studies included the first author, the publication year, study design (cohort or randomised control trial), enrolled sample number in both control and treatment groups, orthopaedic procedure, outcomes regarding the frequency of periprosthetic infection development and the type of antimicrobial coating. The data from each study are summarised in Table 1. The presence of duplicate studies was examined using the Endnote software version X7 (Clarivate Analytics, Philadelphia, Pennsylvania).

### Quality assessment

The methodology of each study was assessed independently by the two senior authors (ODS and PJP) using the Newcastle-Ottawa quality assessment scale 15. Included studies were graded in a three-category scale. Surveys displaying a total score of 0-3, 4-6 and 7-9 were classified as poor, fair or good quality, respectively (Table 2a and Table 2b). A modified Jadad scale for clinical trials was also used to evaluate the quality of included trials 16. A Jadad score greater than 4 was considered to be of high quality. There were no exclusion criteria regarding age, population, diagnosis or quality of the studies. Funnel plots were built in order to determine the aspect of publication bias that may affect the conclusions of our analysis.

### Statistical analysis

Statistical analysis was performed with the MedCalc Meta-analysis Statistical software, version 17.2 (MedCalc Software's, Ostend, Belgium). The incidence of periprosthetic infections after the application of a coated implant and the odd ratios (ORs) and the associated 95% Confidence Intervals (95% CI) were calculated. Heterogeneity between the trials was calculated by using Cochrane *Q* and the inconsistency (*I^2^*) - test. Values greater than 50% were considered as significantly heterogeneous. A random effect model was used to calculate pooled ORs in the case of significant heterogeneity whereas the fixed effect model was used in the studies with low heterogeneity. This was undertaken because in sensitivity analysis the presentation of both models provides comprehensive evaluation of how differences in datasets affected the observed outcomes. Egger's test and funnel plots were used to examine the risk of publication bias. The level of statistical significance was defined as p<0.05.

## Results

### Search results

The literature search and cross-referencing resulted in 1377 references. After the initial evaluation of the studies based on the abstract and title, 111 publications were included. The further analysis of the remaining papers resulted in the exclusion of 59 surveys, as they followed an *in vitro* methodology only. Thirty five studies were excluded because they referred to animal models. Seventeen manuscripts were finally retained 17-33. Based on the inclusion criteria, ten studies were excluded after reading the full article. Finally, seven articles, comparing the incidence of periprosthetic infections between patients with coated implants and controls, were included in our meta-analyses (Table 3). In subgroup analysis, infections after fracture fixation and total replacement were included in the same subgroup not only because total hip arthroplasties could be applied as a primary or revision treatment after hip fractures 34, but it was also noted that osseointegration and bone healing processes share common alterations at molecular and angiogenetic level 35.

According to the Newcastle-Ottawa scale and the modified Jadad score six out of seven of the enrolled trials were considered as of high quality and therefore, were deemed to be at a low risk of bias (Table 2a and Table 2b).

After the evaluation of the funnel plots, all studies were found to lie within a 95% CI as represented by the inverted funnel, suggesting absence of publication bias.

### Study Design and Content

The incidence of periprosthetic infections was reported in seven studies and a total of 1307 patients treated (561 with coating implants and 746 controls without coating implants) were evaluated. Three major coating technologies were analysed. Silver coating was described in three surveys 21, 25, 32, while antibiotic and iodine coating in three 19, 20, 23 and one 17, respectively.

### Silver-coated prostheses

Silver coating of the Modular Universal Tumour and Revision System (MUTARS Implantcast, Buxtehude, Germany) megaprosthesis was carried out by galvanic deposition of 0.91 g (range: 0.33-2.89 g) silver on the surface of titanium prostheses with 10-15mm thickness coating 13. Additionally, low silver-content coating (6 mg) of the Agluna custom-made endoprosthesis (Stanmore Implants Worldwide Ltd, Elstree, UK) was accomplished by ionic silver tacking on the titanium alloy surface after absorption of silver by an aqueous solution 13. Specifically, they were applied after reconstructions due to bone metastasis (N=17) and primary sarcomatous bone tumours (N=77). These prostheses were mainly used after bone tumour reconstruction surgeries. Moreover, MUTARS silver-coated prostheses were applied for arthrodesis revision (N=57) after infected knee replacement surgery 20. Fifteen patients developed periprosthetic or recurrent infections and were treated either by singular irrigation and debridement without implant removal, revision surgery or amputation. None of the patients was diagnosed with leukocyte or liver and kidney impairment and the median silver blood levels were below 200μg/kg, which is considered normal. The causative organisms of post-operation infections were analysed in one study and showed that the leading causes were *Staphylococcus* coagulase negative (N=4), followed by *S. aureus* (N=2), *Pseudomonas aeruginosa* (N=2), *Streptococcus viridans* (N=1) and *Enterococcus faecalis* (N=1) 23.

### Antibiotic-coated prostheses

The most commonly used antibiotic coating was Gentamicin (Table 1). The coating consisted of a fully resorbable poly (D-, L- lactide) matrix (PDLLA) containing gentamycin sulphate 13, applied through dip coating, where the entire nail surface was coated homogeneously. Within the first hour after application, released gentamycin reached 40% of its concentration, approaching 70% and 80% within the first 24 and 48 hours, respectively 13. This coating was applied in Unreamed Tibia Nail (UTN) PROtect and in Expert Tibia Nail (ETN) PROtect, which were based on the original UTN titanium alloy nail (Ti-6Al-7Nb) 13. A novel antibiotic-coating technique, based on the concept of “short-term local implant protection” was DAC (Defensive Antibacterial Coating) hydrogel coating 19, 22. The hydrogel was composed of covalently linked hyaluronan and PDLLA that underwent complete hydrolytic degradation *in vivo* and showed significant antibacterial and antibiofilm activity 21, 25. We must highlight the fact that while implants with PDLLA were coated by a solvent casting technique, DAC hydrogel was directly spread onto the implant by the surgeon before the final insertion of the prosthesis. Cerament (BoneSupport, AG, Lund, Sweden), an injectable synthetic calcium bone substitute composed of calcium sulphate and hydroxyapatite combined with gentamicin (175mg/10mL, Cerament G) or Vancomycin (66mg/mL, Cerament V), was another novel coating technique that was applied in revision joint prostheses re-implantation due to infected primary arthroplasties and demonstrated increased efficacy in infection treatment and osteointegration 24. Cerament was directly applied with a syringe onto the surface of the prosthesis stem 24. Antibiotic coating concerned intramedullary nails and screws-plates for internal fixation in open or closed tibia fractures, in complex revision fracture cases, closed upper and lower limb fractures (N=126) and prostheses for primary (N=189) and revision of infected joint replacements (N=27). Most post-operative infections were superficial at the surgical site. Only six patients developed deep periprosthetic infection or osteomyelitis and were treated with oral or IV antibiotic administration and surgical debridement and nail exchange (N=2) or amputation (N=1) 18, 20, 23. However, these results were based on clinical surveys that analysed the application of DAC hydrogel coating because clinical studies on the poly (D-, L- lactide) with a control group were not identified.

### Iodine-coated prostheses

This type of coating was composed of an electrically produced anodic oxide film and a povidone-iodine electrolyte, resulting in an adhesive porous anodic oxide having the antiseptic characteristics of iodine. The anodic film thickness was between 5 and 10μm, displaying more than 100,000 pores/mm^3^ and supporting 10-12μg/cm^2^ of iodine 27, 29. The iodine coating could be made either on smooth or porous magaprostheses surfaces 27, 29. This type of coating was applied in the tumour endoprosthsesis limb salvage system Kyocera Limb Salvage (KLS) System and KOBELCO K-MAX (Kobelco, Kobe, Japan) and used either for therapeutic or preventive purposes (Table 1). Cases where iodine coating prostheses were applied preventively included endoprostheses for primary or metastatic tumours (N=66). Four patients developed deep surgical site infections and were treated with surgical debridement and oral or IV antibiotic administration (N=3), without implant removal 17.

### Statistical results

**Figure 1:**
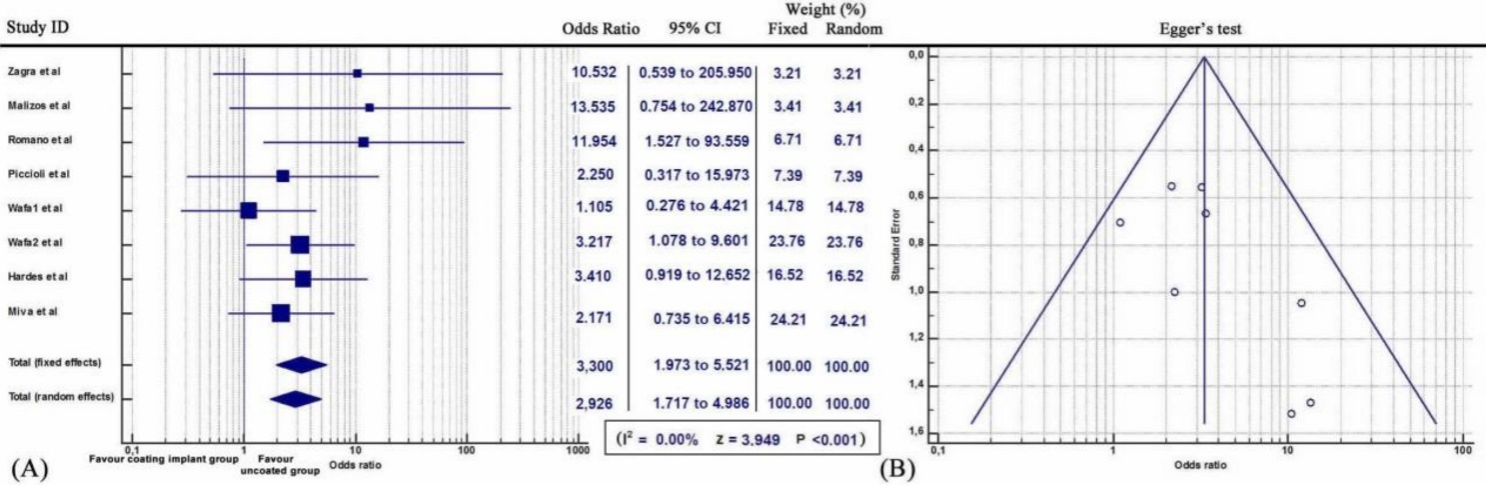
**(A)** Forest plot showing the likelihood of periprosthetic infections in the coating implant group versus the group without coating implants.** (B)** Funnel plot of the Egger's test was utilized to assess for publication bias.

Meta-analysis demonstrated that patients who were treated with antimicrobial coated implants displayed lower infection rate compared with controls over the seven studies (*Q* = 6.1232, *I^2^* = 0.00, 95% CI: 1.717 to 4.986, OR: 2.926, Z= 3.949, p<0.001) (Figure 1).

**Figure 2:**
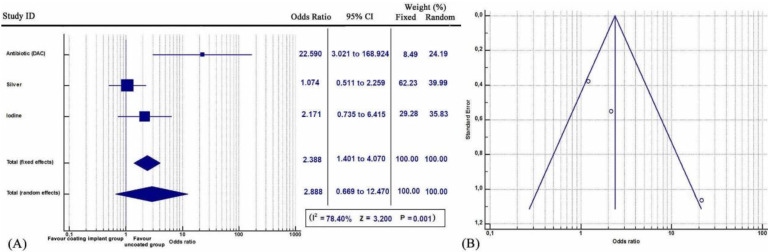
** (A)** Forest plot showing the likelihood of periprosthetic infections in each of the coating implant groups (Antibiotic, Silver and Iodine) versus the group without coating implants. **(B)** Funnel plot of the Egger's test was utilized to assess for publication bias.

**Figure 3:**
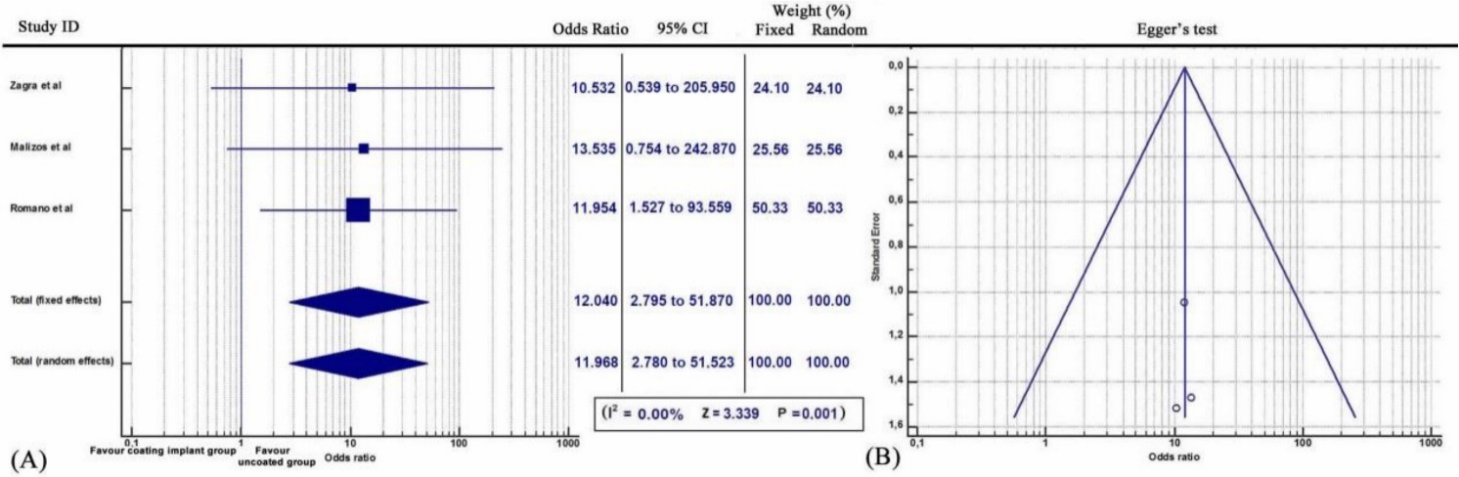
** (A)** Forest plot showing the likelihood of periprosthetic infections in the Antibiotic (DAC) coating implant group versus the group without coating implants. **(B)** Funnel plot of the Egger's test was utilized to assess for publication bias.

**Figure 4:**
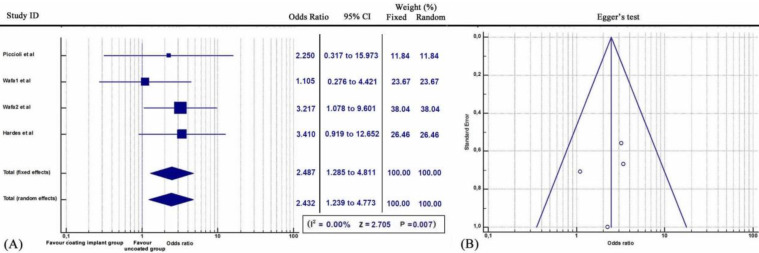
** (A)** Forest plot showing the likelihood of periprosthetic infections in the Silver coating implant group versus the group without coating implants. **(B)**Funnel plot of the Egger's test was utilized to assess for publication bias.

Furthermore, subgroup statistical analysis revealed that each coating technique was effective in the prevention of periprosthetic infections (Q = 9.2606, *I^2^* = 78.40%, 95% CI: 1.401 to 4.070, OR: 2.388, Z= 3.200, p<0.001) (Figure 2). Specifically, both antibiotic coating (Q = 9.2606, *I^2^* = 78.40%, 95% CI: 1.401 to 4.070, OR: 11.968, Z= 3.333, p<0.001) (Figure 3) and Silver coating (Q = 1.7601, *I^2^* = = 0.00, 95% CI: 1.239 to 4.773, OR: 2.432, Z=2.583, p<0.01) were associated with statistically reduced rate of periprosthetic infections (Figure 4).

**Figure 5:**
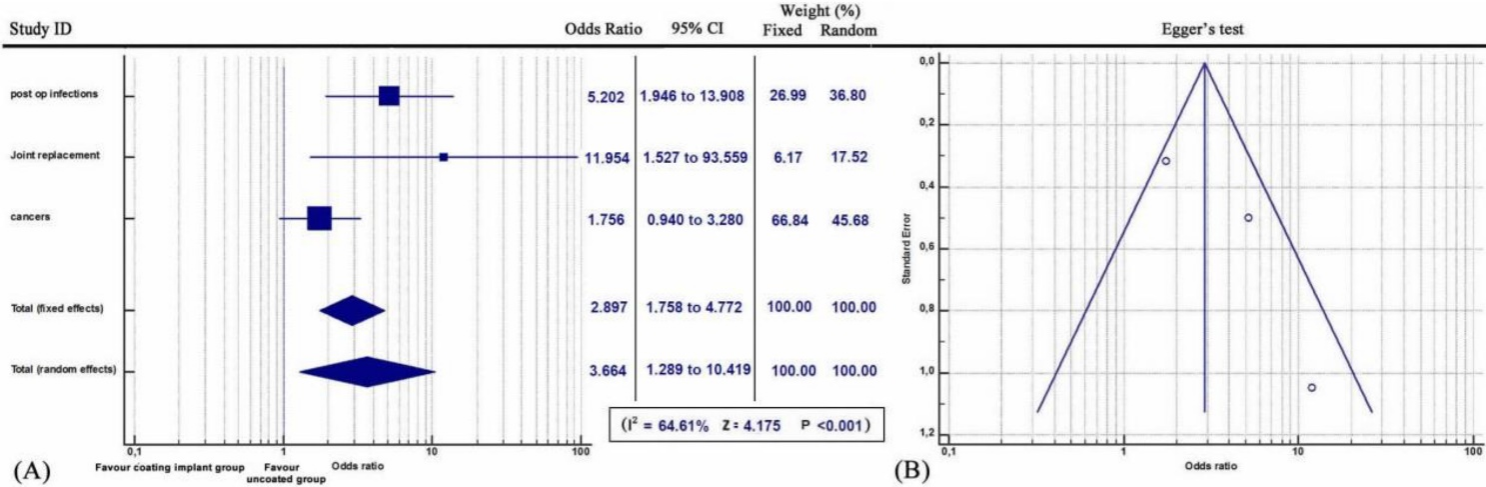
** (A)** Forest plot showing the likelihood of periprosthetic infections including three patients categories (a) with post-operative infections, (b) with primary sarcomatous and metastatic bone cancers and (c) after primary joint replacement arthroplasty in the coating implant group versus the group without coating implants. **(B)** Funnel plot of the Egger's test was utilized to assess for publication bias.

**Figure 6:**
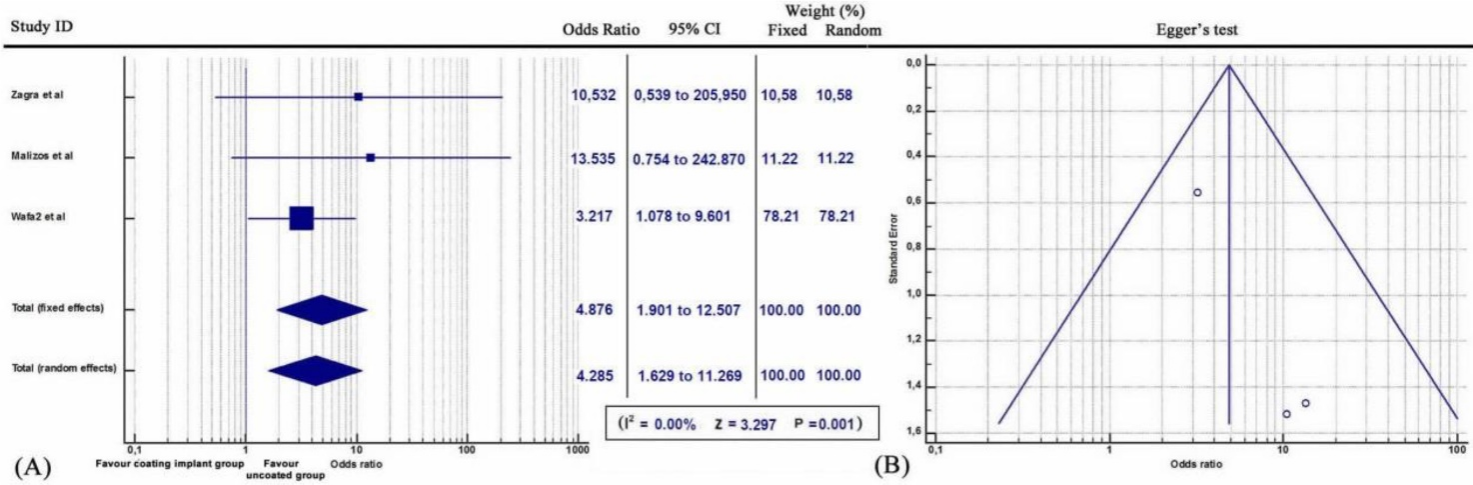
** (A)** Forest plot showing the likelihood of periprosthetic infections in patients suffering of post-surgical infection, in the coating implant group versus the group without coating implants. **(B)** Funnel plot of the Egger's test was utilized to assess for publication bias.

**Figure 7:**
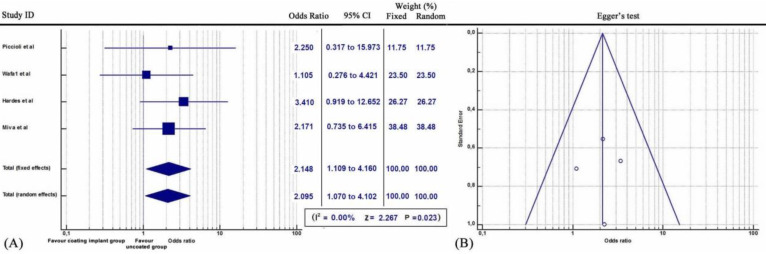
** (A)** Forest plot showing the likelihood of periprosthetic infections in patients suffering of primary or metastatic bone cancers, in the coating implant group versus the group without coating implants. **(B)** Funnel plot of the Egger's test was utilized to assess for publication bias.

Similarly, coating techniques were statistically associated with decreased incidence of periprosthetic infections after their application in patients who underwent revision orthopaedic surgeries due to post-operative infections, cancers and primary joint replacement (Q = 5.6517, *I^2^* = = 64.61%, 95% CI: 1.758 to 4.772, OR: 2.897, Z= 4.175, p<0.001) (Figure 5). In specific, periprosthetic infections in patients who underwent revision orthopaedic surgeries due to post-operative infections (Q = 1.2938, *I^2^* = 0.00%, 95% CI: 1.629 to 11.269, OR: 2.897, Z= 2.949, p<0.001) (Figure 6) and cancers (Q = 1.3623, *I^2^* = 0.00%, 95% CI: 1.070 to 4.102, OR: 2.095, Z= 2.157, p=0.023) (Figure 7) were statistically reduced after the application of coating orthopaedic implants.

## Discussion

To the best of our knowledge, the present systematic review is the first that quantitatively analyses all available data about the current status of orthopaedic implant antimicrobial coating. The main finding of this study was that patients who underwent orthopaedic surgery with coating implants had a decreased risk of post-operative periprosthetic infections. Additionally, subgroup analysis demonstrated that implants with coating techniques were correlated with lowered risk of periprosthetic infections in the cases of reconstruction due to bone cancers, in revision surgeries due to post-operative infections and in primary joint replacements. Although Deng et al conducted a systemic review of surveys on coating techniques; it was more of a qualitative description than a quantitative analysis. Moreover, antibiotic coating cases were not included in the above study 36.

Meta-analysis of clinical data on silver coating showed that silver offers a very good antimicrobial protection and treatment. Specifically, the use of silver-coated prostheses was statistically associated with reduced rates of post-operative or recurrent infections. Furthermore, silver coating was statistically correlated with low incidence of periprosthetic infections in patients who underwent surgical reconstruction for primary or metastatic bone cancers 21, 32 and in the cases of primary and revision arthroplasties due to post-surgical infection 25. The amputation rate also zeroed after use of silver-coated prostheses for knee arthrodesis due to post-arthroplasty complex infection 21. This may be due to the bactericidal activity of silver which that, at low concentrations (35μg/kg), interacted with protein sulphur or phosphorous groups of the bacterial plasma membrane or cell wall (e.g. L-Cysteine) leading to their breakage and bacterial cell death. Silver ions also inhibited cytochromes of the electron transport chain, bound to and damaged bacterial DNA or RNA and increased the production of reactive oxygen species (ROS) that are toxic to bacterial cells 37, 38. These multifunctional actions of silver on different intercellular targets made resistance of the bacterial strains very difficult. The fact that a silver ion are inactivated and unable to develop bactericidal activity when bound to albumin (e.g. haematomas) leads to the elimination of free floating silver ions in the body and it contributes to the very low resistance rate of bacteria. Therefore, routine wound drainage for up to three days after surgical application of silver-coated prostheses was proposed 22. Similarly, the protective effects of silver coating were reduced after 6 months due to its physiological erosion 22. Silver offers an advantage as it is not known to become resistant and its spectrum includes all relevant gram positive and negative bacteria 39-40. In economic terms, although silver-coated implants are more expensive than conventional prostheses, the shorter hospitalisation periods and the decrease in the number of revision surgeries and the additional consequent long-term antimicrobial treatment should also be considered.

Several concerns have been raised regarding the possible toxicities of silver-coated medical devices. An *in vivo* study showed that silver particles promoted inflammatory response, increased osteoclast formation and suppressed bone remodelling 38. A study demonstrated that adverse effects, such as argyria, were negligible. Local argyria manifested as blue to bluish grey skin discoloration in areas exposed to sunlight, due to stimulation of melanocytes by silver. Peripheral neurological deficit due to argyria was observed in two patients 28. No generalised neurological symptoms, ocular argyrosis, raised serum levels of silver or renal and liver toxicities were detected 28. A possible explanation could be that only a small amount of silver is resorbed by the intestine, as most is excreted by the liver. The rest is stored intracellularly, bound to tissue proteins without any functional activity 38. Moreover, local argyria appeared to be an idiosyncratic effect 28.

Antibiotic coating, especially with gentamicin, represented an alternative method of local antibacterial delivery. Our meta-analysis confirmed that gentamycin coating was also a safe technique. Moreover, it was demonstrated that Gentamycin coating (DAC) eliminated the frequency of post-operative infections. Interestingly, gentamycin coating was applied to patients with complex and open fractures, severe soft tissue damage, multiple traumas or late revision cases 19-20, 23. However, the results of this meta-analysis were established by examining clinical surveys of DAC hydrogel coating application. *In vivo* animal models and *in vitro* experiments showed that PDLLA/gentamycin coating prevented bacterial adhesion and proliferation of *S. aureus* and *S. epidermidis* on the implant surface not only preventing biofilm development but also eradicating osteomyelitis even without systemic administration of antibiotics 41. Studies also demonstrated that gentamycin coating did not affect the bone healing process and callus formation, as no differences in fracture healing or in osteoblast function were noted 21, 30. Despite the fact that studies have observed an association between cannulated nails, reaming and increased infection rates 26, 30, 42, high quality randomized trials have not shown a significant difference in the treatment of open fractures between reamed and unreamed nails 43. Although current research studies of gentamycin-coated implants have not shown any gentamycin-associated resistance or nephrotoxic or other side effects, clinicians and researchers are examining new coating approaches with many antibiotic agents like vancomycin or meropenem 19, 20, 23, 26.

Our meta-analysis findings also suggested that iodine-coated implants are effective for the prevention of post-surgical infections in patients suffering from metastatic and primary bone cancers 17. However, only one study with iodine coating was included in our meta-analysis and does not allow us to draw final conclusions. Additionally, the application of iodine-coated prostheses was correlated with a low prevalence of deep and haematogenous infections and it was effective not only for prevention of post-operative infections in tumour patients but also in the treatment of infected joint arthroplasties and pyogenic arthritis 27. Iodine is a broad-spectrum antiseptic covering general bacteria, viruses, bacilli and fungi 28.Finally, Iodine did not cause any drug resistance, was biologically safe as it is excreted by the kidneys and was associated with excellent bone ingrowth and ongrowth properties 27, 29. Side effects after iodine-coated implant use were extremely rare. Allergy to iodine was observed in one patient, without any further clinical or laboratory systemic (e.g. Thyroid) toxic signs.

The limitations of this review were the heterogeneity of data and population, the variability of treatment protocols, the different selection criteria or follow-up periods, the absence of a disease severity classification, the missing statistical analysis of outcomes and recovery rates after application of coated prostheses. Additional limitation factors are the differences in methodological approaches between the studies, the conditions under which the studies were conducted, the absence of the analysis for potential differences of antimicrobial effectiveness based on the type of prosthetic implant and the variation timeline treatment, the type of post-operative antibiotic treatment or the causative microbial agent implicated with post-operative infections, other confounding factors that were not taken into consideration, the fact that the DAC hydrogel studies contributed up to 52% (680/1307) of the patients or the increased risk of bias of the selected studies, especially of those that were not evaluated. We must, also, draw attention to the fact that studies including thoroughly negative assessments about new techniques may face difficulties in publishing by peer-review journals.

## Conclusions

Despite the limitations of our study, our results support the efficacy of antimicrobial coating methods such as silver, gentamycin and iodine, in the reduction of post-operative peri-prosthetic infection rates. Therefore, our meta-analysis suggests that in patients who underwent surgeries for primary or metastatic bone cancers or revision interventions due to post-operative infections and primary total arthroplasties, antimicrobial coating techniques might have positive impact on the prevention of periprosthetic infections. However, further clinical randomised control trials focusing on their antimicrobial characteristics and their adverse events are deemed necessary.

## Figures and Tables

**Table 1:** Clinical data of antimicrobial coated internal, external fixation implements and endoprostheses.

	Author Year	Type of study	Indications/ Surgical interventions	Type of implant	Coating technology	Patients with coating implants (N)	Patients without coating implants (control group) (N)	Periprosthetic infections Coating group(NA) vs Control group: (NC)
1	Miwa et al 2019 17	Retrospective cohort study	Bone metastatic disease, various sarcomas, hemangiopericytoma, adamantinoma of femur,tibia, humerus, pelvis, foot, radius, scapula	Tumour endoprosthesis, joint prostheses, plates, external fixators	Iodine	66	236	NA: 04 NC: 29
2	Zagra et al 2019 19	Prospective randomized cohort study	Revisions for hip arthroplasty infection	Total hip replacement prostheses	Gentamycin, Vancomycin, meropenem (DAC -hydrogel)	27	27	NA: 00 NC: 04
3	Malizos et al, 2017 20	Prospective randomized cohort study	Fresh closed fracture (<7 days) of tibia, knee, femur, humerus, forearm, clavicle, hand	Intramedullary nailing, Screw-plating fixation	Gentamycin, Vancomycin, meropenem (DAC -hydrogel)	126	127	NA: 00 NC: 06
4	Piccioli et al, 2016 21	Retrospective cohort study	Bone metastatic diseases, Various bone sarcomas, giant cell tumour (femur, humerus, tibia, radius, pelvis)	MUTARS tumour endoprosthesis	Silver (galvanic deposition)	17	13	NA: 02 NC: 03
5	Romano et al, 2016 23	Prospective randomized cohort study	Total Joint Replacements	Cementless or hybrid joint prostheses	Gentamycin, Vancomycin Meropenem (DAC -hydrogel)	189	184	NA: 01 NC: 11
6	Wafa et al, 2015 25	Retrospective matched-pair control cohort study	Primary reconstructions, Revisions for infection	Agluna custom- made tumour endoprosthesis	Silver (titanium anodization - aqueous absorption)	85 N1: 26* N2: 59**	85 N1: 24* N2:61**	N1A: 05* N1C:05 N2A:05* N2C:14**
17	Hardes et al, 2010 32	Retrospective cohort study	Various sarcomas, fibrous histiocytoma, giant cell tumour (femur, tibia)	MUTARS tumour endoprosthesis	Silver (galvanic deposition)	51	74	NA: 03 NC: 13

*Follow-up more than 24 months. ** Lost to follow-up rate more than 10% is considered inadequate.

**Table 2a:** Study quality of the studies fulfilling the meta-analysis evaluation criteria based on the Newcastle -Ottawa scale.

	Author Year	Representativeness of the exposed cohort	Selection of the nonexposed cohort;	Ascertainment of exposure	Demonstration that outcome of interest was not present at start of the study	Comparability of cohorts on the basis of the design or analysis	Assessment of the outcome	Follow up long enough for outcomes *	Adequacy of follow-up of cohort **	Total	Quality
1	Miwa 2019 17	1	1	1	1	1	1	0	0	06	Fair
2	Zagra 2019 19	1	1	1	1	2	1	0	1	08	Good
3	Malizos 2017 20	1	1	1	1	2	1	0	1	08	Good
4	Piccioli 2016 21	1	1	1	1	2	1	1	1	09	Good
5	Romano 2016 23	1	1	1	1	2	1	0	1	08	Good
6	Wafa et, 2015 25	1	1	1	1	2	1	0	1	08	Good
7	Hardes 2010 32	1	1	1	1	1	1	0	1	07	Good

*Follow-up more than 24 months. ** Lost to follow-up rate more than 10% is considered inadequate.

**Table 2b:** Study quality of the studies fulfilling the meta-analysis evaluation criteria based on the modified Jadad scale.

	Author Year	Randomization	Concealment of allocation	Double blinding	Total withdrawals and dropouts	Total	Quality
1	Miwa 2019 17	*	*	*	*	04	Good
2	Zagra 2019 19	**	*	*	*	05	Good
3	Malizos 2017 20	**	*	*	*	05	Good
4	Piccioli 2016 21	*	*	*	*	04	Good
5	Romano 2016 23	**	*	*	*	05	Good
6	Wafa et, 2015 25	*	*	*	*	04	Good
7	Hardes 2010 32	*	*	*	*	04	Good

* : indicates one point, **: indicated two points.

**Table 3:**
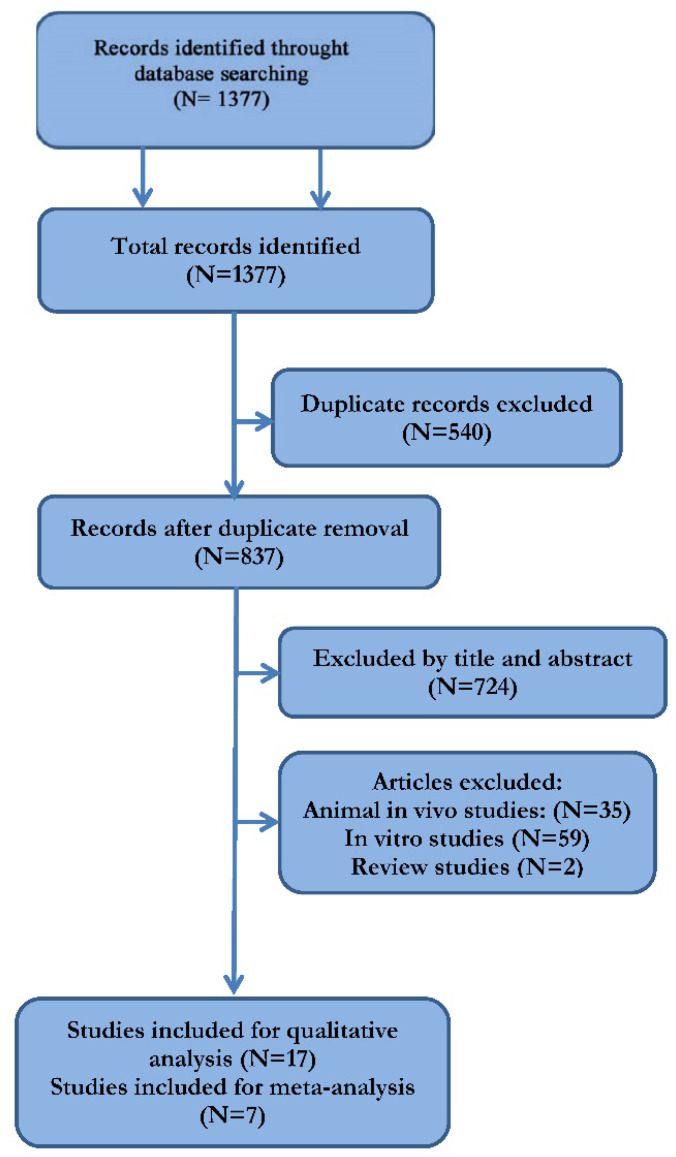
Preferred Reporting Items for Systematic Reviews and Meta-Analysis (PRISMA) flowchart for the searching and identification of included studies.
